# Instantaneous Cardiac Baroreflex Sensitivity: xBRS Method Quantifies Heart Rate Blood Pressure Variability Ratio at Rest and During Slow Breathing

**DOI:** 10.3389/fnins.2020.547433

**Published:** 2020-09-24

**Authors:** Niels Wessel, Andrej Gapelyuk, Jonas Weiß, Martin Schmidt, Jan F. Kraemer, Karsten Berg, Hagen Malberg, Holger Stepan, Jürgen Kurths

**Affiliations:** ^1^Department of Physics, Humboldt-Universität zu Berlin, Berlin, Germany; ^2^Institute of Biomedical Engineering, Technische Universität Dresden, Dresden, Germany; ^3^Division of Obstetrics, Universitätsklinikum Leipzig, Leipzig, Germany; ^4^Potsdam Institute for Climate Impact Research, Potsdam, Germany; ^5^Department of Human and Animal Physiology, Saratov State University, Saratov, Russia

**Keywords:** barorecepter reflex sensitivity, heart rate variability, blood pressure variability, slow breathing, rest

## Abstract

Spontaneous baroreflex sensitivity (BRS) is a widely used tool for the quantification of the cardiovascular regulation. Numerous groups use the xBRS method, which calculates the cross-correlation between the systolic beat-to-beat blood pressure and the R-R interval (resampled at 1 Hz) in a 10 s sliding window, with 0–5 s delays for the interval. The delay with the highest correlation is selected and, if significant, the quotient of the standard deviations of the R-R intervals and the systolic blood pressures is recorded as the corresponding xBRS value. In this paper we test the hypothesis that the xBRS method quantifies the causal interactions of spontaneous BRS from non-invasive measurements at rest. We use the term spontaneous BRS in the sense of the sensitivity curve is calculated from non-interventional, i.e., spontaneous, baroreceptor activity. This study includes retrospective analysis of 1828 measurements containing ECG as well as continues blood pressure under resting conditions. Our results show a high correlation between the heart rate – systolic blood pressure variability (HRV/BPV) quotient and the xBRS (*r* = 0.94, *p* < 0.001). For a deeper understanding we conducted two surrogate analyses by substituting the systolic blood pressure by its reversed time series. These showed that the xBRS method was not able to quantify causal relationships between the two signals. It was not possible to distinguish between random and baroreflex controlled sequences. It appears xBRS rather determines the HRV/BPV quotient. We conclude that the xBRS method has a potentially large bias in characterizing the capacity of the arterial baroreflex under resting conditions. During slow breathing, estimates for xBRS are significantly increased, which clearly shows that measurements at rest only involve limited baroreflex activity, but does neither challenge, nor show the full range of the arterial baroreflex regulatory capacity. We show that xBRS is exclusively dominated by the heart rate to systolic blood pressure ratio (*r* = 0.965, *p* < 0.001). Further investigations should focus on additional autonomous testing procedures such as slow breathing or orthostatic testing to provide a basis for a non-invasive evaluation of baroreflex sensitivity.

## Introduction

The baroreflex is an important component of cardiovascular regulation to maintain homeostasis. The idea of spontaneous baroreflex sensitivity is to estimate the concomitant effect of respiration on heart period and blood pressure based on non-invasive and non-pharmacological driven measurements. Originally, BRS has been assessed by the Oxford method, based on analysis of heart rate response to drug-induced blood pressure variations (Smyth et al., 1969). This method is still the gold standard for assessing baroreflex control. This method has, however, not found wide application in clinical practice due to its laboriousness. It is invasive and requires the administration of vasoactive substances, which is potentially unsafe and costly. Furthermore, undesirable effects of medications on the state of the ANS cannot be excluded. To overcome these drawbacks, numerous methods for non-invasive assessment of BRS have been developed, based on the analysis of spontaneous fluctuations in systolic blood pressure (SAP) and the RR interval (RR). Despite the fact that the idea of using spontaneous heart rate and pressure variations to assess baroreflex may seems desirable, several problems are emerging: In addition to arterial baroreflex itself there are many other sources for pressure and heart rate variations and it is almost impossible to discern baroreflex-driven variations from this mixture. There is no known possibility to isolate specific sets of stimuli and their corresponding reactions. As blood pressure fluctuations during rest in equilibrium are tiny, the effects contributed to the baroreflex seem to be extremely challenging (Lipman et al., 2003) and drastically reducing the signal-to-noise ratio. The synchronous fluctuations of heart rate with respiration, known as respiratory sinus arrhythmia (RSA), are a consequence of the rapid fluctuations of parasympathetic nerve activity toward the sinus node (Blaber and Hughson, 1996). The origin of RSA is known to have various mechanisms (Blaber and Hughson, 1996), including arterial baroreflex and cardiopulmonary baroreceptor responses due to fluctuations of cardiac stroke volume, a direct influence of medullary respiratory neurons on the vagal motor nucleus, and pulmonary stretch receptor response to lung inflation.

Fluctuations in blood pressure and heart period can be of clinical importance as risk markers for cardiovascular morbidity and mortality (Bertinieri et al., 1985; Rothlisberger et al., 2003; La Rovere et al., 2011). However, Lipman et al. (2003) show that spontaneous baroreflex indices do not clearly reflect arterial baroreflex gain. They mainly quantify vagal-mediated heart period oscillations induced by cardiac output fluctuations, and do not reflect barosensory vessel distensibility. Without a clear and consistent relationship the baroreflex gain itself, one can only conclude that spontaneous baroreflex sensitivity cannot be used as proxy for baroreflex gain. Nevertheless, the quantification of this reflex is of great relevance for understanding the cardiovascular system and for risk stratification (Bertinieri et al., 1985; Rothlisberger et al., 2003; La Rovere et al., 2011). Recently (Wessel et al., 2020) we were able to show that the spontaneous sensitivity of the arterial baroreflex (BRS) under resting conditions cannot be estimated by the sequence method (SME), which only quantifies the quotient of heart rate and systolic blood pressure variability. In this paper we test whether the xBRS method (Westerhof et al., 2004; Wesseling et al., 2017) is suitable to quantify the baroreflex sensitivity from non-invasive, non-interventional measurements under resting conditions. Therefore, two surrogate analyses were performed in which, due to the design, no causal relationships between blood pressure and heart rate signal can be present. Furthermore, SME and xBRS were calculated from data collected not only under resting conditions but also under controlled breathing.

## Data

To allow comparison to the results in Wessel et al. (2020), we reanalyzed the same data from 5 different studies in obstetrics, genetics, cardiology and heart surgery (Faber et al., 2004; Barantke et al., 2008; Retzlaff et al., 2009; Boyé et al., 2011; Retzlaff et al., 2011) in a similar manner. Demographic data of all sub-studies were given in Wessel et al. (2020): “All patients gave written, informed consent, and all studies were approved by the respective local ethics committees. From obstetrics (Faber et al., 2004) 915 measurements of 304 pregnant women were included (mean age 28.4 ± 5.4 years). The data contain 398 recordings of healthy women, 120 from patients with chronic hypertension, 38 from gestational hypertension, 152 from women who later developed pre-eclampsia, 88 from pre-existing hypertension with pre-eclampsia, 12 with other hypertensive disease and 78 from women with intrauterine growth restriction. From genetics (Barantke et al., 2008) we considered measurements from 367 subjects with an age of 10 to 88 years (45.0 ± 16.3 years), 157 were male (43%). From cardiology (Boyé et al., 2011) we used the measurements from 75 patients with chronic cardiac diseases referred for primary preventive implantable cardioverter-defibrillator implantation following Multicenter Automatic Defibrillator Implantation Trial study criteria, mean age 70.9 ± 10.1 years, body mass index 27.0 ± 3.5. From Retzlaff et al. (2009) 302 measurements from patients before and after aortic (AV) or mitral valve (MV) surgery were included for analysis. The mean age of the AV patients and MV patients was 62 ± 13 years and 59 ± 2 years, respectively. From Retzlaff et al. (2011) 169 measurements from 58 consecutive patients undergoing either trans-catheter aortic valve implantation (TAVI) or surgical aortic valve replacement (SAVR) with the heart-lung machine and being in stable sinus rhythm were enrolled. Thirty four of them underwent SAVR and 24 of them TAVI, 28 males, mean age 64.6 ± 13.8 in the SAVR group and 80.5 ± 7.3 in TAVI.

All measurements of the considered studies were performed under supine resting position for 30 min using the Task Force Monitor (CNSystems, Graz) or the PortaPres device (Finapres Medical Systems, Enschede). In total we gathered 1,828 time series containing the beat-to-beat values of heart rate (HR) as well as systolic blood pressure (SBP). Exclusion criteria were atrial fibrillation, pacemaker activity, technical artifacts, as well as ectopy time greater than 10%, reducing the number of time series to 1,576 – careful visual inspection for further technical and physiological artifacts reduced the subjects for reanalysis to 1,439.

In addition to this comprehensive data set of rest measurements, recordings with certain autonomous testing procedures were analyzed in this paper. We used 245 measurements of 44 healthy pregnant women, mean age 30 ± 5.4 years, from the Fetal Autonomic Cardiovascular rEgulation (FACE) study which is currently in progress at the University of Leipzig Medical Center in cooperation with the TU Dresden and the Humboldt-Universität zu Berlin. The study was approved by the committee of ethics of the University of Leipzig Medical Center (357/17-ek). One aim of this study was to characterize the reaction of fetal autonomic regulation to maternal paced breathing based on a context dependent biosignal analysis. The measurements were performed under supine resting position using the PortaPres device (Finapres Medical Systems, Enschede), the fetal ECG signal was recorded from the abdomen of the pregnant woman. Our measurement protocol included 10 min of measurement at rest in supine position, 5 min of paced slow respiration (period 7.5 s – 8 respiration cycles per min), and 5 min of fast respiration (period 3 s – 20 cycles per min). Between both paced respiration conditions was one break of 5 min at rest. Exclusion criteria were rhythm disturbances (many of ventricular or supraventricular ectopic beats), technical artifacts, and incomplete study protocols. The data underwent a careful visual inspection for further artifacts by experts which reduced the analyzed data set to 184 records.

## Materials and Methods

Originally, BRS was measured by injecting vasoconstrictive agents to raise blood pressure, i.e., quantifying the reflex-like increased beat-to-beat intervals in the electrocardiogram (ECG). Later, attempts have been made to determine baroreflex sensitivity non-invasively, most often using spontaneous heart rate variability (HRV) and blood pressure variability (BPV) obtained from continuous finger pressure measurement (Bertinieri et al., 1985; Rothlisberger et al., 2003). The underlying hypothesis is that there is always some spontaneous variability in blood pressure that should allow an estimate of BRS. In this paper, the BRS estimation method under consideration was the xBRS method (Westerhof et al., 2004; Wesseling et al., 2017).

xBRS is a time domain method designed for estimation of BRS from non-invasively obtained SBP and beat-to-beat-interval (BBI) data. The original time series is resampled to obtain evenly sampled data at 1 Hz. Instead of sequences, xBRS uses sliding windows with a fixed length of 10 seconds. For every SBP window, there should exist at least one corresponding BBI window (in a lag range of 0–5) with positive and significant cross correlation (*p* < 0.05, two sided test for zero correlation). The BBI window with highest cross correlation is then selected. xBRS has two advantages: (a) it gives more valid “sequences” for the same time series compared to SME and (b) it allows to observe slow regulation circuits that are presumably arising from sympathetic control. Usually, the resulting number of valid “sequences” in the time series is large enough to enable the calculation of an “instant” measure for xBRS. The xBRS method was developed by Westerhof et al. (2004) and initially it set strict constrains on p-value of the cross correlation (*p* < 0.01). In the last revision (Wesseling et al., 2017), the threshold for the *p*-value was increased to 0.05, it doubles the percentage of necessary valid windows. Once sequences are selected, the ratio between the standard deviations of BBI and of SBP is used as a measure of baroreflex sensitivity. The reasoning for this decision was that the measure based on ratios of standard deviations does not differ significantly from the slopes of the regressions but is computationally more efficient. In our article we implemented the last revision of the xBRS method; the geometric mean of the instant xBRS values was used as BRS estimate per study period reflecting the approximately log-normal distribution of xBRS values (Wesseling et al., 2017).

Furthermore, different parameters from the time domain were calculated to quantify short term HRV and BPV in this large data set (Wessel et al., 2020, cf. Table 1). The RMSSD_RATIO_ defined as RMSSD_HRV_ divided by RMSSD_SBP_ showed the highest correlation to the sequence method for BRS estimation there (Wessel et al., 2020).

**TABLE 1:** Basic characteristics of considered parameters in the final data set (Mean ± *SD*: mean value ± standard deviation) and the correlation coefficient r to xBRS (*R* to xBRS, *p* < 0.001 for all coefficients).

	Mean ± *SD*	R to xBRS
xBRS [ms/mmHg]	8.4 ± 5.3	1
xBRS_S1_ [ms/mmHg]	8.4 ± 5.2	0.99
xBRS_S2_ [ms/mmHg]	7.9 ± 4.7	0.97
meanNN [ms]	761 ± 146	0.54
meanBP [mmHg]	129 ± 22.4	−0.2
sdNN [ms]	42.5 ± 18.6	0.67
sdBP [mmHg]	8.1 ± 2.9	−0.25
RMSSD [ms]	25.6 ± 14.7	0.84
RMSSD_SBP_ [mmHg]	2.9 ± 1	−0.21
RMSSD_RATIO_ [ms/mmHg]	9.5 ± 6.1	0.94

*xBRS_S1_, surrogate 1 of xBRS; xBRS_S2_, surrogate 2 of xBRS; meanNN, mean beat-to-beat-interval of HR; meanBP, mean blood pressure of SBP; sdNN, standard deviation of HR; sdBP, standard deviation of SBP; RMSSD, root mean square of successive differences of HR; RMSSD_SBP_, root mean square of successive differences of SBP; RMSSD_RATIO_, Ratio of rmssd and rmssd_sbp.*

**FIGURE 1:**
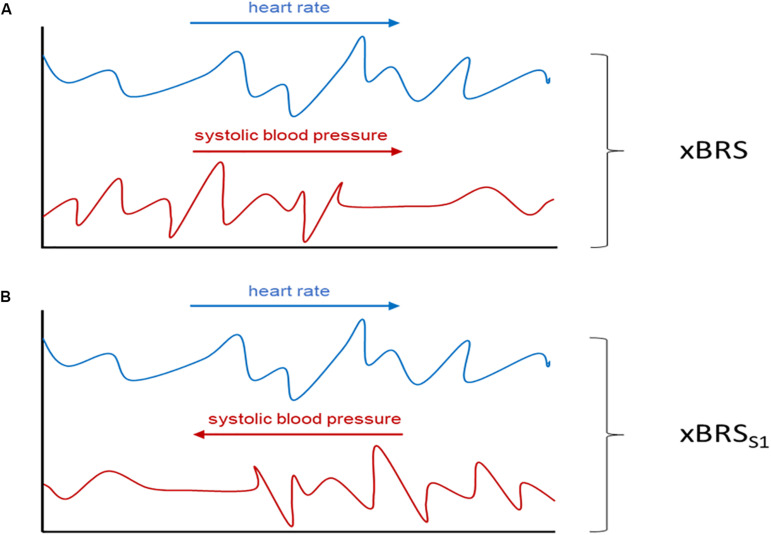
Schematic illustration of the performed surrogate analysis. In **(A)** xBRS is calculated from the original heart rate (blue) and the systolic blood pressure series (red) while in xBRS_S1_ analysis the systolic blood pressure series is time reversed **(B)**. Adapted from Wessel et al. (2020).
(b) Beat-to-beat-intervals were shuffled using the IAFFT approach (Schreiber and Schmitz, 1996). By applying this procedure causal relationship between heart rate and blood pressure has, again, been removed (xBRS_S2_).

Analogous to (Wessel et al., 2020) we performed two surrogate analyses in order to ascertain whether xBRS can quantify causal relationships between heart rate and blood pressure and thusly spontaneous BRS:

(a) Systolic blood pressure time-series were analyzed in reversed order, i.e., the first blood pressure value is now the last, the second now the second last etc. This results in surrogate data with the same distribution as the original data since the values of each point are the same, just in a different time position. However, any causal relationship between heart rate and blood pressure has been removed by this procedure (cf. Figure 1).

Both surrogate tests are used to test the following hypothesis: “xBRS does quantify causal relationships between heart rate and blood pressure.”

Statistical analysis was performed using IBM SPSS Statistics version 24. To quantify significant relations between the parameters used in this study we applied Pearson’s correlation as a measure of the linear relationship between two continuous random variables. This measure does not assume normality, but assumes finite variance as well as covariance - properties which were assumed for our data sets.

## Results

**FIGURE 2:**
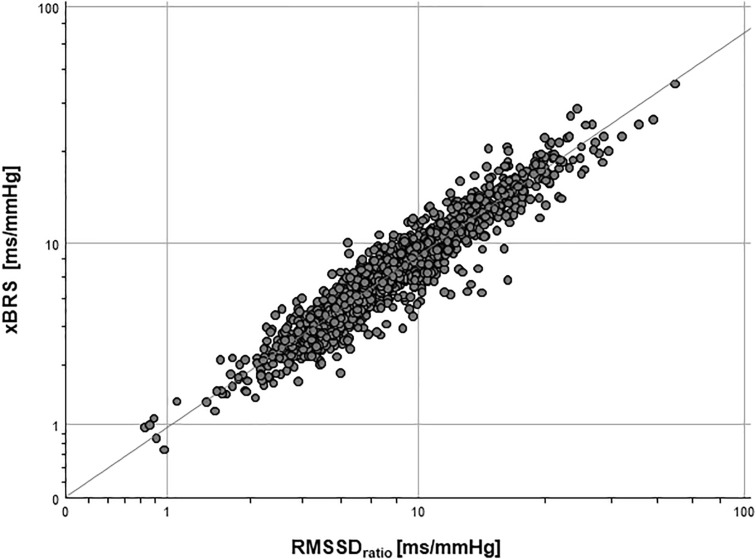
Log-log-plot of xBRS and RMSSD_RATIO_: *R*^2^ = 0.898, *p* < 0.001.

Table 1 shows the results of the correlation analysis between xBRS and further HRV and BPV parameters in the final data set (*n* = 1,439). The highest correlation coefficient r was found between xBRS and short-term variability parameters RMSSD (*r* = 0.84, *p* < 0.001). A more precise estimate yields the highest correlation coefficient for RMSSD_RATIO_ (*r* = 0.94, *p* < 0.001, cf. Figure 2).

**FIGURE 3:**
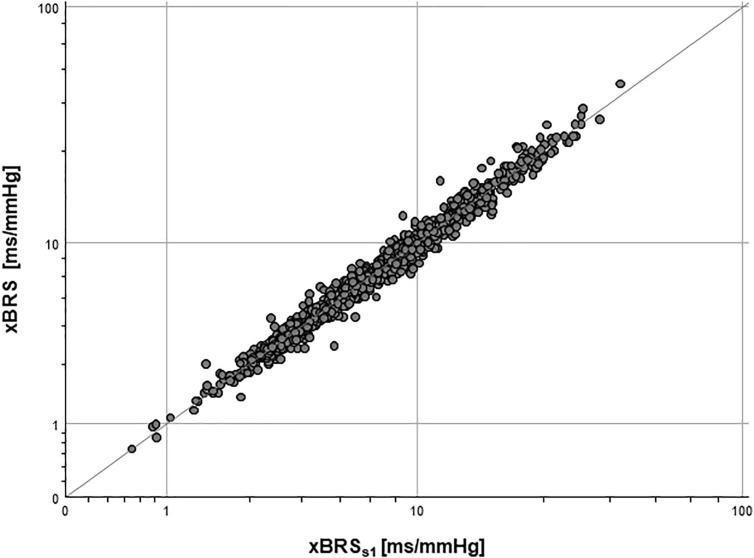
Log-log-plot of original xBRS and surrogate xBRS_S1_: *R*^2^ = 0.979, *p* < 0.001.

Surrogate analysis (a) between xBRS_S1_ and RMSSD_HRV_ showed high correlation coefficient (*r* = 0.84, *p* < 0.001), i.e., reversing one time series does not affect the results of xBRS. Moreover, the correlation coefficient between xBRS_S1_ and RMSSD_RATIO_ was equally high with *r* = 0.94, *p* < 0.001. Finally, the correlation coefficient between original xBRS and surrogate xBRS_S1_ was extremely high with *r* = 0.99, *p* < 0.001 (cf. Table 1 and Figure 3).

In the surrogate analysis (b) the correlation coefficient between xBRS_S2_ and RMSSD_HRV_ was *r* = 0.84, *p* < 0.001. Moreover, the correlation coefficient between xBRS_S2_ and RMSSD_RATIO_ was equally high with *r* = 0.93, *p* < 0.001. Finally, the correlation coefficient between the original xBRS and surrogate xBRS_S2_ again was extremely high with *r* = 0.97, *p* < 0.001 (cf. Table 1).

To overcome concerns for bias due to the presence of multiple measurements per subjects, we repeated our procedures on a reduced data set with only the first measurement per subject (*n* = 733) and got similar correlations (xBRS vs. RMSSD: 0.83, xBRS vs. RMSSD_RATIO_: 0.93, xBRS_S1_ vs. RMSSD: 0.83, xBRS_S1_ vs. RMSSD_RATIO_: 0.93, xBRS vs. xBRS_S1_: 0.98). We conclude that the comparison of time series with different time bases has no influence on the results of xBRS. xBRS as an estimate for the spontaneous BRS shows a potentially large methodological bias. This contradicts the hypothesis that xBRS at rest quantifies causal relationships between heart rate and blood pressure.

**FIGURE 4:**
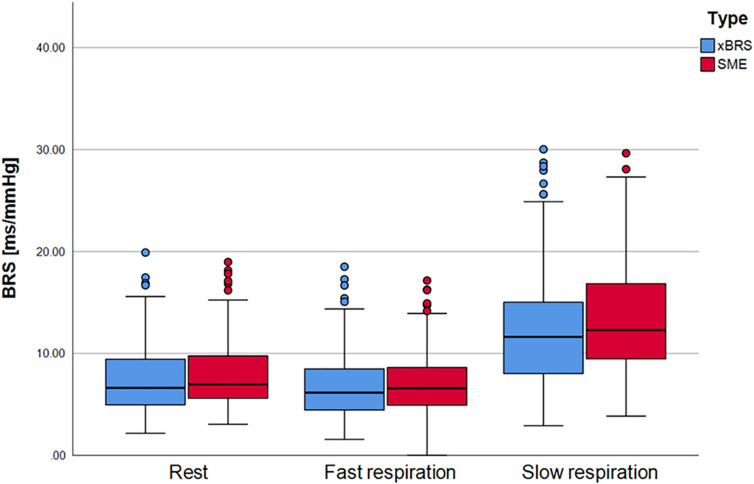
xBRS and SME as BRS estimates for the FACE study. During rest and fast respiration procedure there are only small differences. xBRS and SME values for slow respiration, however, are statistically significant higher as for resting and fast respiration.

In order to investigate whether any causal relationship could be quantified by xBRS under slow breathing conditions, we analyzed the data from the FACE study. Figure 4 shows the xBRS and the SME values under rest conditions, under rapid breathing as well as under slow breathing. There is a, clearly significant, increase in xBRS and SME with slow breathing (*p* < 0.001), showing that measurements at rest only involves certain range baroreflex activation, but not the full capacity of the arterial baroreflex. However, these estimates are, again, exclusively dominated by the heart rate - systolic blood pressure ratio (*r* = 0.965, *p* < 0.001). Moreover, after performing the surrogate analyzes described above, the xBRS estimates do not change between original and surrogate data: The correlation coefficient at rest: xBRS to xBRS_S1_ *r* = 0.98, to xBRS_S2_ *r* = 0.96; 3 s (fast) breathing: xBRS to xBRS_S1_ *r* = 0.98, to xBRS_S2_ *r* = 0.94; slow respiration: xBRS to xBRS_S1_ *r* = 0.99, to xBRS_S2_ *r* = 0.97.

In summary, we found that, even under controlled breathing conditions, no causal relationship between beat-to-beat intervals and the systolic blood pressures could be quantified by xBRS.

**FIGURE 5:**
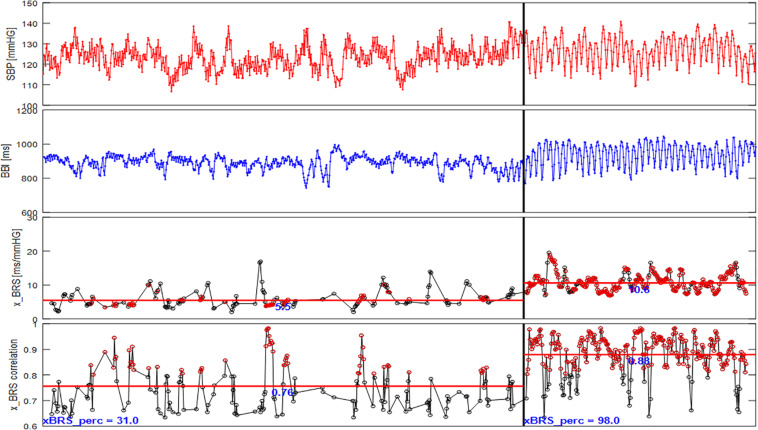
xBRS results for one subject of the FACE study (SBP, systolic blood pressure; BBI, beat-to-beat intervals; x_BRS; x_BRS correlation, the correlation coefficient at valid epochs). During rest (left panel) xBRS value is on average 5.5 ms/mmHg, the average correlation at valid epochs is 0.76, the percentage of valid epochs is 31% only. The xBRS values for slow respiration (right panel) is on average 10.6 ms/mmHg, however, the average correlation at valid epochs is 0.88 and the percentage of valid epochs is 98%. The red markers in the lower panels mark cross correlation values higher than 0.8.

Nevertheless, Figure 5 shows that significantly more, as well as more consistent, results are obtained under controlled slow breathing compared to normal resting measurements. While under resting conditions only 31% of all calculated correlations are significant for this example and are included in the calculation of the xBRS, during slow breathing, the percentage of valid xBRS windows increases to 98%. Furthermore, the mean correlation between heart rate and systolic blood pressure windows is significantly higher during slow breathing. Taken together, these values support the hypothesis that the xBRS values at slow breathing are more consistent with the BRS. A higher BRS implies a higher regulatory capacity, meaning large blood pressure fluctuations can be balanced well by BBI changes which in turn supports maintenance of homeostasis. However, a low baroreflex sensitivity can lead to deviations from homeostasis and thus to events such as a hypertensive crises or fainting spells due to low blood pressure.

## Discussion

In this paper we test the hypothesis that the xBRS method quantifies the causal interactions of spontaneous BRS from non-invasive, non-interventional measurements at rest. For the surrogate analysis we substituted the systolic blood pressure by its reversed time series and thusly removed their causal relationship. Our analysis showed that xBRS remains unchanged. Therefore, we conclude that xBRS is not able to estimate causal relationship between heart rate and systolic blood pressure. Our results of the surrogate analysis show, that xBRS can be mostly explained by the short-term HRV, quantified by the RMSSD. Minor deviations of this univariate model are adequately explained by the simple bivariate model RMSSD_RATIO_ which is also based on non-causal interactions.

In contrast, La Rovere et al. (1998) was able to show that low values of pharmacologically determined baroreflex sensitivity (pBRS < 3 ms per mmHg) carry a significant risk of cardiac mortality after myocardial infarction. This indicates a difference between the xBRS estimation at rest and an invasive pBRS, which not only refers to respiration-induced fluctuations, but also includes carotid extensibility (La Rovere et al., 2011). We suspect that the estimation of baroreflex sensitivity is unreliable in cases of relatively shallow breathing. In these cases, there is also only a small respiratory induced blood pressure variation, which leads to only small baroreceptor activation and thus to a low HRV. Even then, in cases where any BRS estimation would result in a spurious low result, the baroreflex could still be fully functional and its sensitivity in the normal range. The BRS could just be impossible to estimate using the currently dominant protocol, i.e., relaxed respiration in resting supine position (Lipman et al., 2003). Goldstein et al. (1982) and Lipman et al. (2003) show that baroreflex sensitivity varies greatly from patient to patient and that different mechanical (neck chamber) and pharmacological techniques for measuring baroreflex sensitivity are likely to measure different aspects of baroreflex function. This contradicts the idea of spontaneous baroreflex sensitivity in the sense that the sensitivity should not be affected by the way it is being measured. Recently developed sophisticated methods for BRS estimation disentangle the effects of respiration from heart period and blood pressure (Pinna et al., 2015; Maestri et al., 2017; Bari et al., 2019), however, all methods fail to be reliable estimates of BRS. An increase in SME values during slow breathing lead Tzeng et al. (2009) to systematically review the baroreflex function. His hypothesis was that controlled slow breathing, which causes higher blood pressure fluctuations, increases cardiovagal baroreflex gain in young healthy subjects. Baroreflex enhancement was investigated using both the classical pBRS and the non-invasive SME method. Compared to breathing at rest, slow breathing was associated with a significant increase in the SME index, while the pBRS remained unchanged. The SME values for slow breathing are higher than the pBRS values in the study, which could be a result of overestimation or a systematic error in the pBRS determination. However, Arica et al. (2011) showed that pBRS values can be predicted from non-pharmacological indices acquired during slow breathing. From both studies we derive the opinion that autonomic testing should allow a reliable, non-invasive, non-pharmacological driven quantification of the baroreflex gain. This will require large scale medical studies, where the BRS is measured invasively according to the state of the art [modified Oxford method (Ebert and Cowley, 1992)] and additional runs of autonomous tests are performed.

To investigate whether a causal relationship between heart rate and blood pressure exist under controlled breathing conditions, we analyzed the data from the FACE study. We found a clearly significant increase in xBRS and SME under slow breathing conditions compared to rest or fast breathing, showing that the latter ones involve only limited baroreflex activity. However, for all conditions these estimates are exclusively dominated by the heart rate – systolic blood pressure ratio. Moreover, after performing the surrogate analyzes, the xBRS does not vary between original and surrogate data. Thus, even under controlled breathing conditions, no causal relationship between beat-to-beat intervals and the systolic blood pressures could be found.

In conclusion, we demonstrated for all short measurements, under resting conditions and controlled breathing, that RMSSD_RATIO_ carries similar vagally mediated information as xBRS. However, we found, under controlled breathing, a potentially large methodological bias in xBRS and SME as estimates for the baroreflex sensitivity. During slow breathing estimates for SME and xBRS are significantly increasing, which clearly shows that measurements at rest are only accompanied by limited baroreflex activity, but not to the full capacity of the arterial baroreflex. Further investigations should focus on additional autonomic testing procedures (e.g., orthostatic test, carotid occlusion, neck suction) to provide a better empirical foundation of non-invasive assessment of the baroreflex sensitivity.

## Data Availability Statement

The data that support the finding of this study are fully available to the authors but cannot be shared publicly due to risk of violating privacy. Requests to access results datasets should be directed to NW.

## Ethics Statement

The studies involving human participants were reviewed and approved by the University of Leipzig Medical Center (357/17-ek). The patients/participants provided their written informed consent to participate in this study.

## Author Contributions

NW, AG, KB, JKr, and JKu contributed conception and design of the study. NW, HM, and HS organized the database. AG performed the statistical analysis. NW wrote the first draft of the manuscript. All authors contributed to manuscript revision, read and approved the submitted version.

## Conflict of Interest

The authors declare that the research was conducted in the absence of any commercial or financial relationships that could be construed as a potential conflict of interest.
